# Social Interactions Receive Priority to Conscious Perception

**DOI:** 10.1371/journal.pone.0160468

**Published:** 2016-08-10

**Authors:** Junzhu Su, Jeroen J. A. van Boxtel, Hongjing Lu

**Affiliations:** 1 Department of Psychology, University of California Los Angeles, Los Angeles, CA, 90095, United States of America; 2 School of Psychological Sciences, Faculty of Medicine, Nursing and Health Sciences, Monash University, Clayton Campus, Victoria, 3800, Australia; 3 Department of Statistics, University of California Los Angeles, Los Angeles, CA, 90095, United States of America; Brain and Spine Institute (ICM), FRANCE

## Abstract

Humans are social animals, constantly engaged with other people. The importance of social thought and action is hard to overstate. However, is social information so important that it actually determines which stimuli are promoted to conscious experience and which stimuli are suppressed as invisible? To address this question, we used a binocular rivalry paradigm, in which the two eyes receive different action stimuli. In two experiments we measured the conscious percept of rival actions and found that actions engaged in social interactions are granted preferential access to visual awareness over non-interactive actions. Lastly, an attentional task that presumably engaged the mentalizing system enhanced the priority assigned to social interactions in reaching conscious perception. We also found a positive correlation between human identification of interactive activity and the promotion of socially-relevant information to visual awareness. The present findings suggest that the visual system amplifies socially-relevant sensory information and actively promotes it to consciousness, thereby facilitating inferences about social interactions.

## Introduction

The human ability to deal with social stimuli enables us to recognize what others are doing and to understand why others act in certain ways, so that social interactions can be planned and executed. How the human visual system processes social stimuli is a fundamental question in social perception, as this mechanism is the basis for understanding of the goals and intentions of others [1]. Biological motion perception is a prime example of this type of processing [2]. Previous research has shown that sparse motion stimuli, consisting of just a few point-light dots representing joint movements of a human actor, can be readily analyzed to recognize detailed characteristics of the actor. These characteristics include gender, identity, action category, emotion and interpersonal interaction [3–8]. Moreover, such simple displays with sparsely distributed moving dots suffice for inferences about the goals and beliefs of other actors [8, 9]. Hence, the faculty of action perception and understanding entails a rather direct bridge between sensory representations and social cognition.

However, the complexity of the social world constantly challenges the capacity of the human visual system. Social scenes usually are cluttered with many actions involving different people and objects (e.g., imagine a scene in a busy train station or an airport). Deep processing of all actions and movements in the sensory input is difficult to achieve due to the limited capacity of the human visual and cognitive system. This inherent constraint may have entailed mandatory competition among observed actions for conscious experience. A central unresolved question in social cognition concerns how sensory information important for social inference is processed within the visual hierarchy [10]. In the present study, we investigated whether the brain amplifies socially-relevant information and actively promotes it to consciousness, when observing human actions and inter-personal interactions, so as to facilitate social perception and inferences.

Previous research has demonstrated top-down influences of action perception on sensory processing. For example, recognizing meaningful actions biases depth estimation toward an interpretation consistent with human body structure, even when the physical depth is inconsistent with the layout of human body [11, 12]. Such findings suggest that knowledge about the structure and dynamics of human body movements guides the interpretation of sensory information in visual stimuli. Recent evidence also indicates that the presence of interpersonal interactions enhances sensitivity to detecting actions when the point-light stimuli are embedded in a noise background [6, 8]. This finding suggests that coordinated body movements between two actors impact on how actions of an individual actor are processed. These studies used action stimuli with relative short durations (range of 1 to 8 seconds). However, social interactions between agents in daily life extend for much longer periods. Over extended time, the availability of socially relevant stimuli to visual awareness may fluctuate, especially when other competing dynamic information in the visual scene requires access for processing. It remains unknown whether social actions and interactions receive priority to conscious perception compared to other non-social dynamic information.

There are reasons to anticipate that actions engaged in social interactions may receive priority to visual awareness. First, due to the importance of interactive activities in inferring social relations between agents, it would be beneficial for the organism to maintain higher sensitivity to such stimuli than to non-interactive actions. Indeed, previous research showed that certain brain areas (in particular, the medial parietal and dorsomedial prefrontal cortices) are jointly recruited to yield increased activity when observing social interactions [13]. Second, it has previously been shown in binocular rivalry displays (i.e. displays in which the two eye’s inputs compete for subjective awareness) that contextual cues play important roles in determining which stimulus reaches conscious awareness. For example, when two gratings are presented in the two eyes, each eye’s view is dominant for approximately equal amounts of time. However, when one of the two stimuli is embedded in a larger display, this global context causes it to be more visible [14, 15]. These studies showed that contextual information biases the selection processes of sensory input at an early stage of visual analysis. This process may also apply to social stimuli. Indeed, in social situations involving two interacting people, a rich set of contextual cues becomes available, originating from coordinated movements and a common goal. In these situations, the actions of one actor dictate, to a high degree, the actions performed by the companion actor. These contextual cues may play an important role in selecting and promoting action information to conscious perception.

Therefore we adapted a binocular rivalry paradigm to examine the impact of social action stimuli on some of the earliest stages of visual processing. When the two eyes receive markedly dissimilar patterns, observers experience the phenomenon of binocular rivalry: at any moment in time one of the patterns is perceived as the dominant stimulus, but perception alternates between the rival patterns every few seconds [16]. These fluctuations afford a sensitive measure of the impact of stimulus variables (e.g., contrast) or object-based properties on rivalry dynamics [17], thereby shedding light on how the brain constructs our visual perceptions.

When two action stimuli, each consisting of a dozen disconnected point-lights, are presented to different eyes and put into conflict in a binocular rivalry paradigm, one might expect that these disconnected dots would undergo rivalry independently of each other. However, a previous study showed that the point-lights representing a walking action tended to undergo rivalry as a grouped entity [18], suggesting that the visual system uses knowledge about human body movements as a way to structure the input.

Here we examine whether social interactive activities, as opposed to solitary actions from a single individual (e.g. walking), impact the rivalry between two competing action stimuli to determine what action information reaches awareness and what information does not. In the present study, salsa dancers were used to generate rivalry stimuli, taking advantage of the rich whole-body movements in the social action sequence and the high coordination of movements between the two dancers. We expected that actions eliciting natural body movements that are involved in meaningful interpersonal activities would be granted preferential access to visual awareness over actions that are not involved in interpersonal activities, when measuring visual dominance in binocular rivalry displays.

## General Methods

The study was approved by IRB #12-000277-CR-00004. Consent forms and debriefing documentations were provided. Stimuli were created using the Psychophysics Toolbox [19, 20] and were displayed on a calibrated Viewsonic CRT monitor with a resolution of 1280 × 1024 pixels. Participants viewed the stimuli through an adjustable stereoscope from Berezin Stereo Photography Products in a constant viewing distance of 57 cm maintained by a chin rest. All actions were obtained from the Carnegie Mellon Graphics Lab Motion Capture Database (http://mocap.cs.cmu.edu). The BioMotion Toolbox [21] was used to convert the raw motion capture files to the point-light display.

On each trial two rival point-light actions were presented, one to each eye and in different colors (i.e., blue and red) to induce binocular rivalry. The rival actions were displayed in the same retinal region of the two eyes for a relatively long duration in the range of 20 to 26 seconds. Actors subtended 7.3 by 5.3 degrees of visual angle. Point-lights were shown with luminance level of 10.6 cd/m^2^ for all colors (red, green or blue) on a black background (~0 cd/m^2^). The size of each point-light was 0.13°. A central fixation cross (with size of 0.75° by 0.75°) was presented, as well as a frame surrounding the stimuli on the screen to help subjects register the same position in the two eyes. The size of the frame was 13.28×13.28° with a line width of 0.075°. In order for the rival actions to spatially overlap as much as possible to create sufficient rivalry perception, we eliminated extrinsic motion of the body by fixing the mid-point of the two hip joints of the rival actors at the same position across the entire trial.

**Fig 1:**
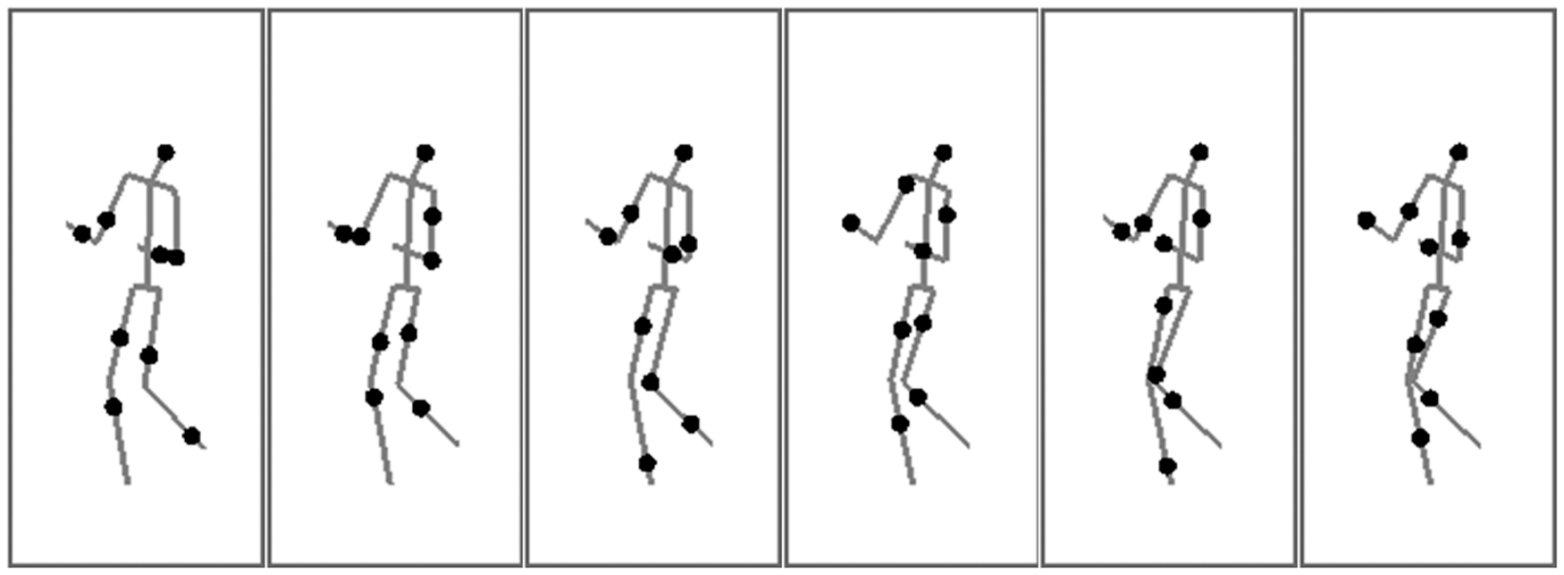
An illustration of stimulus frames presented in the limited-lifetime display. Dots were randomly sampled along the limbs and were displayed for one frame. The lines are shown just for the purpose of demonstration.

Previous research [22, 23] has shown that motion signals with speed around 1.2 deg/s strongly attract dominance in rivalry, yielding exclusive visibility of one eye’s view [24]. To rule out the possibility that the perceived speeds of smoothly moving joints determine the rivalry dynamics, we randomly sampled point-lights along the limbs, with a limited lifetime of one frame using the method developed by Beintema and Lappe [25]. Previous research showed that humans still can readily recognize actions presented in the limited-lifetime display. This method removes the smooth motion trajectories of displayed dots, thereby eliminating potential contributions from inter-frame local motion signals in binocular rivalry, allowing us to focus on effects attributable to action processing based on posture change over time. As shown in Fig 1, point-lights were randomly sampled along the limbs and were displayed with a limited lifetime of one frame (i.e., 13ms). Each limited lifetime actor was composed of nine dots, including the head and eight dots randomly sampled on each of the 8 limb segments.

For all the experiments reported in the present paper, participants were asked to press and hold one of three keys to indicate whether the “blue” (left arrow) or “red” (right arrow) dots were more visible, or the two groups of colored dots were equally visible (down arrow), at any moment throughout the trial.

We excluded participants who failed to satisfy two criteria: (a) over all trials combined, an observer pressed a specific response button (corresponding to red or blue rival action) more than 95% or less than 5% of the time; (b) there were more than 16 trials (corresponding to 50% trials in Experiment 1) in which an observer only pressed the button corresponding to “mixed” percepts during the entire trial. These criteria applied to all experiments.

### Experiment 1: Natural activities are more visually dominant than inverted activities

Previous research reported that two upright point-light walkers, each with a different facing direction, elicited stronger rivalry than two inverted walkers [18]. This finding suggests that actions with more ecologically-relevant upright orientation are granted a preferential access to visual awareness. This rivalry result is consistent with the well-documented inversion effect in the literature on biological motion perception [26]. However, when an upright action is placed in direct conflict with an inverted action, it is still unknown whether the upright action will gain visual dominance and/or the inverted action will be suppressed. Experiment 1 aims to address this question by measuring visual dominance when displaying an upright action to one eye, and an inverted action to the other eye. This experiment examined whether the rivalry inversion effect of a familiar action (e.g. walking, [18]) can be generalized to relatively unfamiliar actions (e.g., dancing), and whether this effect is still observed when limited lifetime dots (i.e. each point change positions randomly on the limb across frames) removes inter-frame motion trajectory information. We predicted that the upright dancer would receive preferential access to awareness (i.e. stronger predominance in rivalry) when competing with an inverted dancer.

#### Methods

Participants completed a practice session using the walking rival stimuli as in Watson, Pearson and Clifford. [18]. The practice session included four trials showing two upright walkers with different facing directions (leftward or rightward) presented one to each eye, in different colors (red or blue), and four extra trials showing inverted walkers as the rival stimuli, in intermixed order. Participants were asked to press and hold a button to indicate the color of the dominant dots (red, blue or mixed) throughout the 26-second trials.

**Fig 2:**
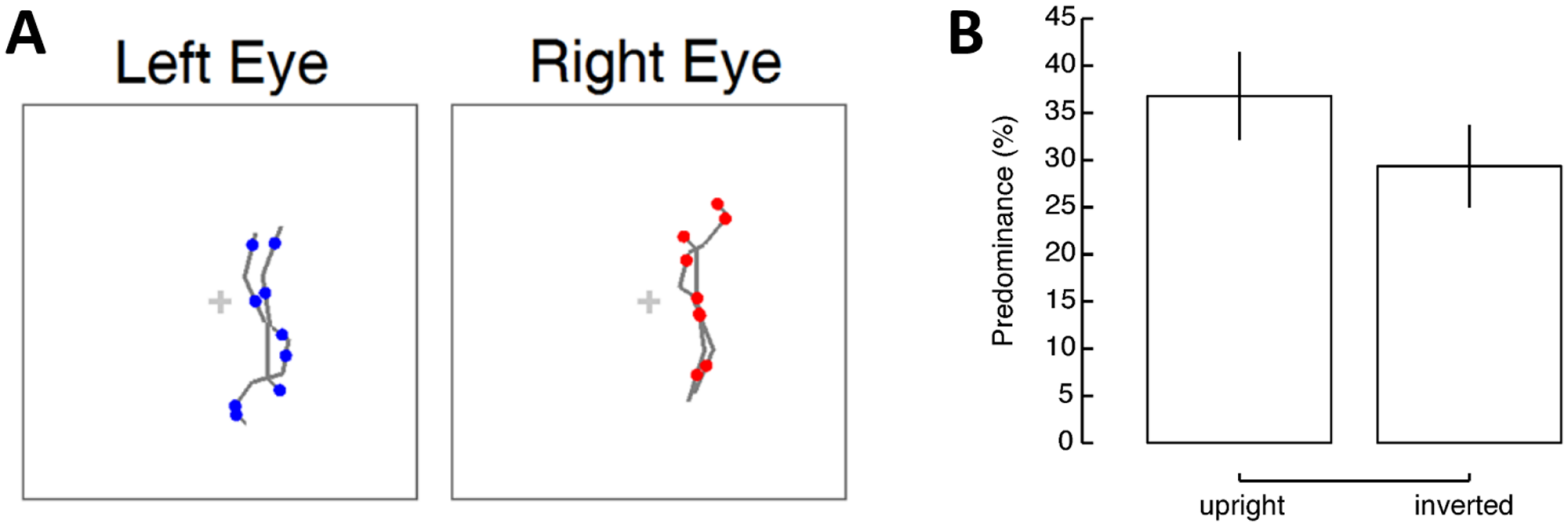
Stimulus illustration and results in Experiment 1. (A) Schematic illustration of rival actions in different colors in dichoptic presentation. One eye viewed an upright dancer and the other eye viewed an inverted dancer. The gray lines are only for illustration purpose to show the randomly sampled dots along the limbs, and were not shown in the experiment. (B) Results of Experiment 1 are presented as predominance, for both upright and inverted dancers. Error bars indicate the 95% confidence interval throughout the paper.

In each trial of the subsequent test session, one of eight female salsa dancers was selected to generate both rival actions, one upright and one inverted (see Fig 2A). The stimuli were presented dichoptically: an upright dancer to one eye and an inverted dancer to the other eye, each in a different color. The rival actions were displayed with a different random sampling of the limited lifetime point-lights. An inverted dancer is often still recognizable as human activity but with much lower recognition accuracy. For example, Dittrich [27] found that recognition accuracy for inverted waltz dancer was 61% in a free response task. Hence, inverted dances serve as a social but less ecological stimulus in rivalry. Participants were asked to indicate the color of the dominant actor (red, blue or mixed) continuously throughout the entire trial. The rival stimuli lasted for 26 seconds on each trial. The experiment consisted of 32 trials for each of the upright and inverted conditions.

#### Participants

All participants were undergraduate students at the University of California, Los Angeles (UCLA) and participated for course credit. All observers had normal or corrected-to-normal visual acuity. Sixteen observers (13 female, average age of 20.7) participated in Experiment 1. One participant’s data were excluded from the analysis based on the above described exclusion criteria. The sample size was estimated according to relevant studies on binocular rivalry with social stimuli in the literature [18] and pilot studies. The number of around 20 subjects is consistent with the sample size used in binocular rivalry studies if using naïve subjects. The exact number of participants was determined by a stopping rule of convenience, in that we stopped collecting data at the end of the week in which we achieved as least 20 participants (this was less in experiment 1, based on the cited literature).

#### Results

The predominance (the percentage of reported dominance over the total viewing duration) for upright dancers (*M* = 36.81%) was significantly greater than that of the inverted dancers (*M* = 29.35%; *t*(14) = 2.43, *p* = .029, Cohen’s *d* = .83; see Fig 2B). We also examined a second measure of binocular rivalry, the average time duration of visual dominance for each rival stimulus. We found that upright dancers were visible for longer durations than inverted dancers (upright, *M* = 4.49 s; inverted, *M* = 4.13 s; *t*(14) = 2.30, *p* = .037), providing converging evidence that actions with the more ecologically-relevant upright orientation receive precedence to visual awareness compared to inverted actions.

#### Discussion

The present results bolster and extend the findings reported by Watson et al.[18] (2004). Our results suggest that when upright and inverted actions directly compete with each other in a rivalry setup, the visual system employs the ecologically-relevant upright body orientation as a reference to group the visual input into meaningful and coherent units, even for dancing actions that observers do not regularly perceive or perform in daily life. The present experiment employed the limited-lifetime technique to alleviate the potential contribution of local motion mechanisms, such as a “life-detector” based on characteristic movements of the feet in recognizing and detecting walking actions [28, 29]. Hence, the present paradigm allows us to identify rivalry effects primarily attributable to global action processing.

### Experiment 2: Interactive activities are more visually dominant

Experiment 2 aimed to examine whether an action engaged in social interaction receive precedence in access to consciousness compared to a solitary action performed by a single actor. Binocular rivalry was created by presenting a male salsa dancer to one eye, and a non-salsa actor to the other eye. We predicted that without showing the interacting partner, the two rival actions (salsa dancer and non-salsa actor) would show comparable visual dominance during rivalry. On the contrary, when presenting a binocularly-viewed actor performing a dance with one of the rival actors (i.e., in the presence of social interaction), this social salsa dancer would receive preferential access to visual awareness compared a non-interactive actor.

#### Methods

**Fig 3:**
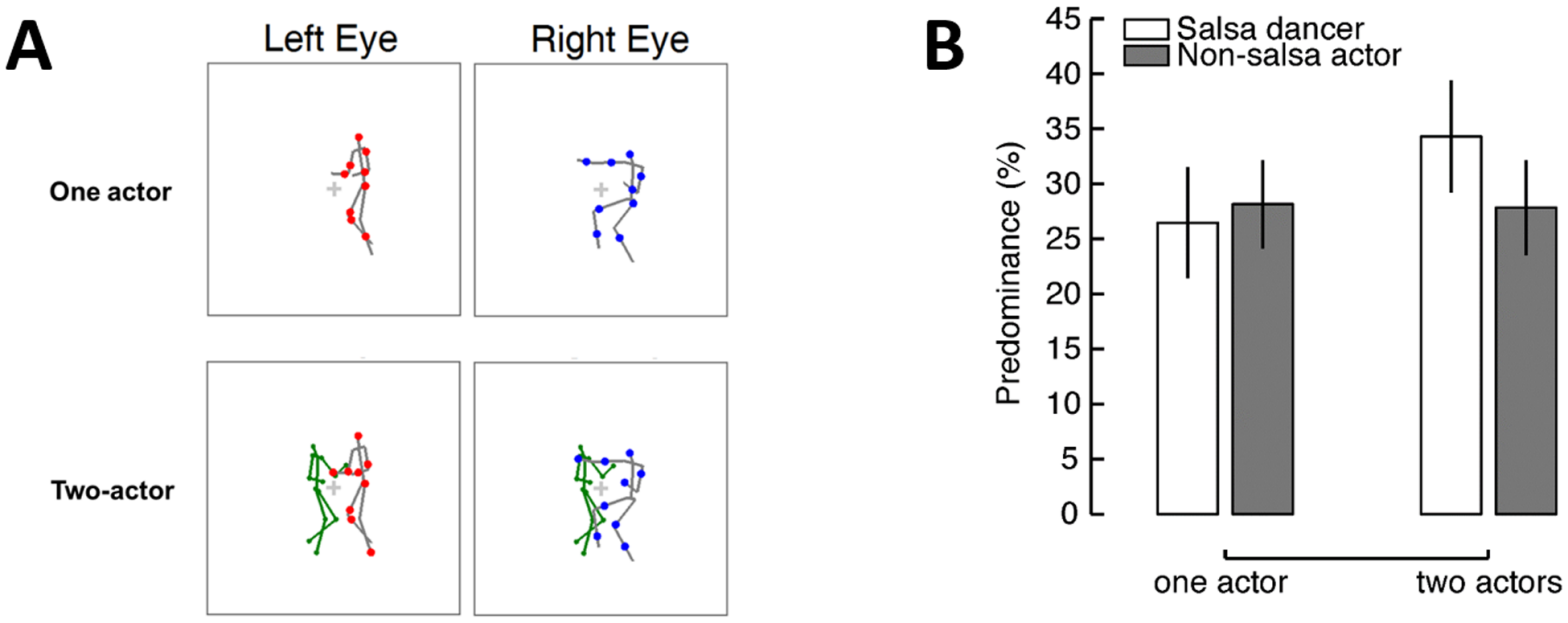
Stimulus illustration and results in Experiment 2. (A) Schematic illustration of a frame from the rival stimuli in dichoptic presentation. The partner of the rival salsa dancer was binocularly presented in green in the two-actor condition, and was absent in the one-actor trials. The two rival actions were shown in a limited-lifetime display (the gray lines are only for illustration purpose). In this illustrated stimulus at the bottom, the two actors shown to the right eye did not engage in a meaningful interaction, whereas the two actors presented to the left eye performed a salsa dance (a meaningful interaction between two actors). (B) Results of Experiment 2. The predominance difference between the two rival actions was significantly greater in the presence of the partnered dancer than in its absence.

In Experiment 2, the rival dichoptic stimuli were presented in a limited-lifetime display, one in red and the other in blue (counterbalanced across trials). The rival stimuli consisted of a male salsa dancer, randomly selected from one of the four salsa couples, and a non-salsa actor (an exuberant laugher or an Indian dancer; see Fig 3 for an illustration). The rival non-salsa actions included most characteristic movements of the salsa dance, such as arm waving, leg lifting, and body shaking. We ensured that low-level stimulus characteristics, such as the average inter-frame speed of joint movements, and the size of the actors, were matched between the rival salsa dancers and the competing non-salsa actors. S1 Fig depicts the summary of the matched average inter-frame speeds between the rival actions used in the experiment. We thus minimized the potential difference of low-level visual features in the two rivalry stimuli during binocular rivalry. In the *one-actor* condition, a single actor was presented to each eye including the male salsa dancer and the non-salsa actor to provide the rivalry actions. In the *two-actor* condition, the stimuli included the same rivalry actions as those in the one-actor condition but also with a third actor presented to both eyes in the left side of the visual field. This binocularly-viewed non-rival action was generated from a female salsa dancer that was partnered with the male salsa dancer at the time when the actions were recorded, thus displaying a truly inter-personal activity. As shown in Fig 3A panel, the binocularly-viewed non-rival action was shown as a green stick figure with the line width of 0.038 visual degrees to minimize the effort needed to recognize this partner dancer. The rivalry stimuli lasted for 20 seconds in each trial. The experiment consisted of 64 trials. As in Experiment 1, participants were asked to indicate the color of the dominant actor (red, blue or mixed) at any moment throughout the entire trial.

#### Participants

26 observers (22 female, average age of 20.6) participated in Experiment. No observers participated in more than one experiment in the present paper. According to the exclusion criteria, five participant’s data were excluded in the analysis.

#### Results

As shown in the Fig 3B, when the binocularly-viewed dance partner was absent, the two rival actions did not differ in visual predominance (male salsa dancer, *M* = 26.46%; non-salsa actors, *M* = 28.15%, *t*(20) = 0.99, *p* > .250), indicating that there was no inherent visual dominance preference for the salsa dancer over the non-salsa actor. The equal visibility of the two rival actions indicates that matching of low-level stimulus characteristics between the two actions during binocular rivalry was successful.

However, in the presence of a binocularly-viewed partner dancer, the rival salsa dancer became more dominant, receiving precedence to visual awareness compared to the rival actor who did not engage in a social interaction. This result is supported by a significant two-way statistical interaction effect (*F*(1,20) = 8.34, *p* = .009, $\eta_{p}^{2}$ = .29) in a repeated measures ANOVA on predominance with two within-subject factors, rival action types (salsa dancer/non-salsa action) and interactivity (one-actor/two-actor). We also found that the significant increase in predominance of the salsa dancer when the partnered dancer was displayed compared to when the partnered dancer was not displayed (presence, *M* = 34.30%; absence, *M* = 26.46% *t*(20) = 3.60, *p* = .002, Cohen’s *d* = .66).

Additional analyses on the average duration of visual dominance, provided converging evidence; the difference of the dominance durations between the two rival actors depended on the presence of the partnered dancer (*F*(1,20) = 10.33, *p* = .004, $\eta_{p}^{2}$ = .34). Specifically, when the partnered salsa dancer was displayed (despite being viewed binocularly), the dominance duration for the rival salsa dancer significantly increased compared to that when the partnered dancer was absent (5.54s with partner vs. 4.33s without partner, *t*(20) = 2.69, *p* = .014, Cohen’s *d* = .33). The consistent findings from both predominance and dominance duration suggest the existence of a mechanism that boosts priority for promoting interactive actions to visual awareness. This mechanism does not act on non-interactive actions, as there was no evidence of increased suppression for non-interactive actions.

### Experiment 3: Inversion reduces the impact of interactive activities on visual dominance

It might be argued that the rivalry behavior in Experiment 2 is driven by certain mid-level visual features, rather than social interpersonal activity. Such mid-level features include symmetry or movement coordination between the partnered salsa dancers. These critical features could serve as informative cues to trigger the mechanisms of grouping the two dancers, and consequently render the interactive action more visible. This impact could conceivably take place without the identification of the social content contained in action stimuli. In order to address this issue, Experiment 3 was designed to include two within-subject sessions, one showing upright actions and the other with inverted actions. The inversion manipulation significantly weakens effective identification of action and interactive activity [4, 6, 26], but maintains mid-level features such as symmetric postures and coordinated dot movements between partnered dancers, and other potential grouping cues in the stimuli with interactive activity.

#### Methods

Experiment 3 included one session identical to Experiment 2, and the other session in which the actors were inverted in all trials. The order of the two sessions was counter-balanced across participants. The inversion manipulation reduces holistic action processing [4, 6, 26], which consequently weakens the percept of interactive activity, while maintaining motion cues and inter-personal coordination identical to that in the upright condition. 26 observers (17 female, average age of 21.2) for Experiment 3. All participants satisfied the inclusion criteria and were included in the analysis.

#### Results

First, Experiment 3 replicated the findings of Experiment 2 for the predominance data for the upright actions, indicated by a significant two-way interaction effect between the interactivity (one-actor/two-actor) and rival action type (salsa dancer/non-salsa action) (*F*(1,25) = 11.37, *p* = .002, $\eta_{p}^{2}$ = .31). This finding confirms that the predominance difference between the rival actions with ecological body orientation (i.e. upright display) depended on the presence of interactive activity in the display.

When the actions were inverted, the impact of interactive activity on the predominance difference was reduced to trend level (*F*(1,25) = 4.18, *p* = .052, $\eta_{p}^{2}$ = .0.14). This marginally significant result appears to suggest that coordinated movements between two actions may play a role in determining the predominance measure even if the actions are displayed upside-down. However, we found an influence of the block order (inverted session first or upright session first) on predominance, revealed by a significant four-way interaction effect (*F*(1,24) = 6.055, *p* = .021) in a mixed ANOVA analysis with the order of the blocks as a between-subjects factor, and orientation (upright/inverted), interactivity (one-actor /two-actor) and rival action type (salsa dancer/non-salsa action) as within subjects factors.

**Fig 4:**
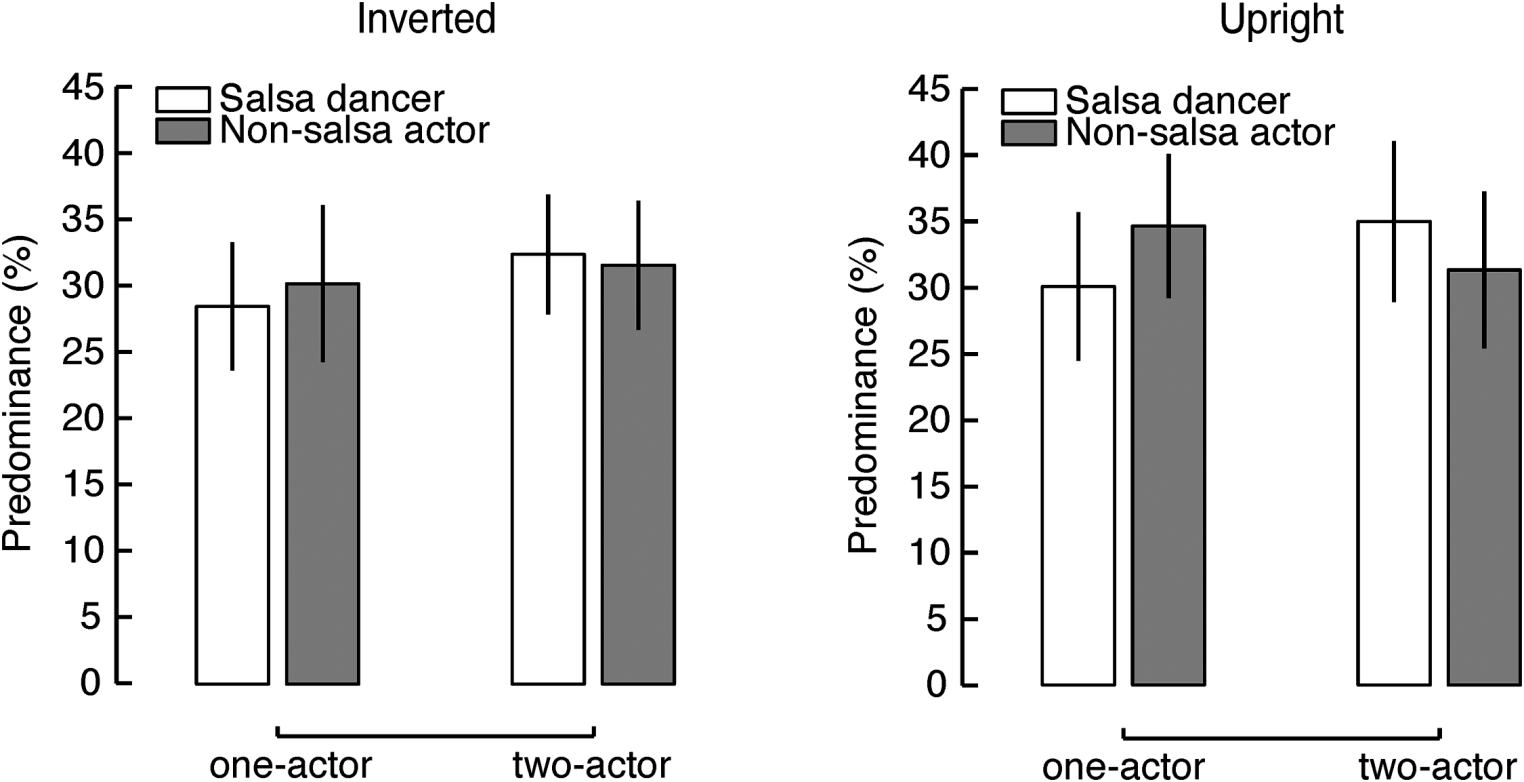
Results of Experiment 3. Predominance values for the participants who performed the inverted block (left) before the upright block (right).

Inversion only caused the social interaction effect to disappear when the inverted session was run first (Fig 4). This result suggests that the influence of inversion was susceptible to a learning effect when observers familiarized themselves with coordinated salsa dancing in a prior block with the upright display. This learning effect is consistent with previous findings that humans appear to be able to “learn” to see inverted action. For example, Hiris and his colleagues found that people can learn to detect the presence of inverted biological motion nearly as well as they detected upright actions [30]. When focusing only on the participants who performed the inverted block first, and hence lacked the learning opportunity of improving recognition with inverted actions, we found that the impact of interactive activity on the predominance difference was significant in the upright condition (*F*(1,13) = 9.28, *p* = 0.009, $\eta_{p}^{2}$ = 0.42), but not in the inverted condition (*F*(1,13) = 0.99, *p* = 0.34, $\eta_{p}^{2}$ = 0.07). These results suggest that, without prior exposure to point-light displays, coordinated movements and symmetry cues preserved in the inverted condition were not sufficient to elicit a significant influence on visual dominance of interactive actions.

Similarly, we found that the difference of the dominance durations in upright rivalry depended on the presence of the partnered dancer, as indicated by a significant two-way interaction effect (*F*(1,25) = 7.94, *p* = .009). In contrast, the same effect was not significant (*F*(1,25) = 2.72, *p* = .111) in the inverted condition. These results suggest the impact of interactivity on maintaining visual dominance was reduced when rival actions were presented upside-down, likely due to the weakened perception of inverted actions.

#### Discussion for Experiment 2 and 3

The findings in Experiment 2 from two binocular rivalry measurements (i.e. percentage of total dominance and average duration of each dominance) show that social interactions presented in the upright body orientation propel relevant actions into conscious awareness. Results in Experiment 3 further suggest that inverted displays of the actions reduced such advantage for the social interactions to enter conscious awareness.

Hence, the gating of social actions into awareness may be most effective when interactive activity is apparent or easy to extract from visual inputs with ecological body orientations (i.e., upright). In the visual world, social interactions usually involve two agents coordinating their body movements. Coordinated motion can be a strong predictor of interactive activity. Hence, consistent with findings supporting the critical role of movement coordination in joint action [31, 32], perception of coordinated body movements might enable the promotion of potential socially-relevant information to visual awareness to a certain degree, even when social interaction appears difficult to perceive in some situations (e.g., the inverted display in our paradigm, when viewed after a block of upright displays).

### Experiment 4: Identification of interactive actions promotes visual dominance

Identifying the presence of an interactive activity with upright actions was quite effortless in Experiment 2 and 3, because the partnered salsa dancers were either absent or present. It has been suggested that when actions are simple and goal-directed, the analysis can be performed by an automatic process [33]. However, more complicated tasks, such as identifying inter-personal interaction and inferring social intentions, require attention [34–37]. In Experiment 4 we increased the difficulty of the tasks by introducing the third binocularly-viewed salsa dancer in all conditions (i.e. partnered vs. un-partnered). We also engaged attention by explicitly asking participants to identify whether two agents were performing meaningful interactive activities besides performing the primary task of tracking the visual rivalry. We predicted that partnered dancers would receive more visual dominance in rivalry compared to un-partnered dancers when attention is explicitly demanded in discriminating genuine inter-personal interactions from two actors performing similar actions independently without coordination.

#### Methods

**Fig 5:**
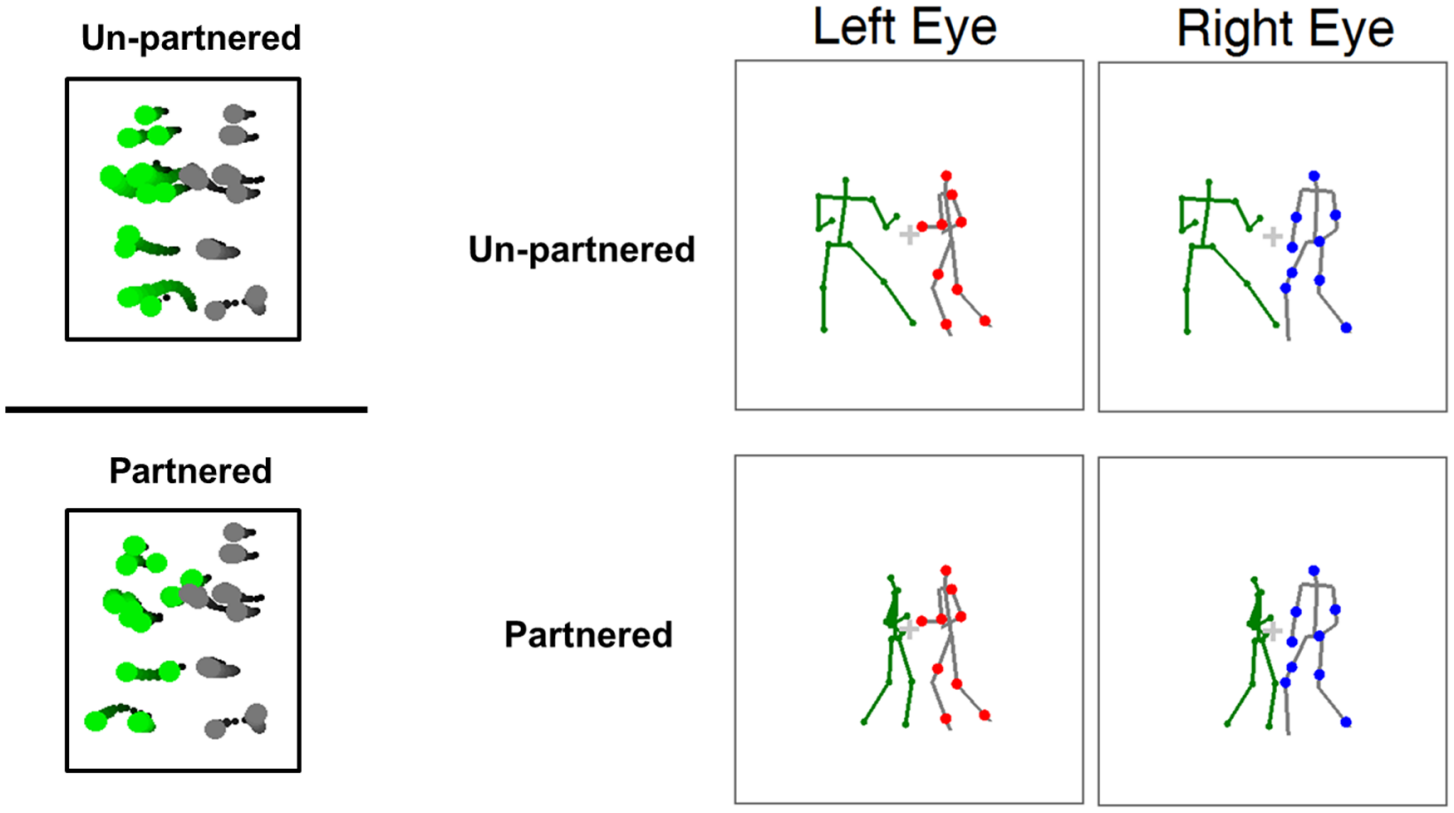
Schematic illustration of stimuli used in Experiment 4. (A) The trajectories of joint movements for a pair of un-partnered two male salsa dancers and a coupled salsa pairs. To give an impression of movement, this schematic shows several frames with increasing dots sizes for more recent frames. In this example, the two dancers in the un-partnered condition did not engage in a coordinated activity, whereas the two dancers in the partnered condition performed a salsa dance (a meaningful interaction between two actors). (B) The stimuli included two rival actions displayed in red and blue, and a binocularly viewed action displayed in green.

Experiment 4 used the same rival stimuli as in Experiment 2: a male salsa dancer presented to one eye, and a non-salsa actor (either a laughing person or an actor performing an Indian dance) to the other eye. A third actor was shown to both eyes as a green stick figure. In the *partnered* trials, the third actor was the female partner dancer engaged in a salsa dance with the rival male dancer so as to form a dynamically-coupled interpersonal interaction. In the *un-partnered* trials, the third actor was a male salsa dancer from one of the other recorded dance couples, thus sharing a similar action style and statistical regularity of kinematics with the rival salsa dancer, but not engaging in interactive dancing with the rival male actor. An alternative way of removing the synchronicity between agents (which indicates interactivity) is to split the action sequence into two sequences and cross-pair the sequence of each individual actor, as was done in a previous study [6]. We did not use this manipulation because some of the recorded dancing actions were not long enough (i.e. longer than 40 seconds) to allow minimal trial durations of 20 seconds. Fig 5 illustrates the rival stimuli and the trajectories in the two conditions of this experiment.

Participants were asked to indicate the color of dominant dots during binocular rivalry, with the same instructions as in the first two experiments. In addition, at the end of each trial, they reported whether any two actors in the display had performed interactive activities. This second task was designed to engage attention to social interactions between agents in the observed actions. Experiment 3 consisted of 64 trials, each lasting for 20 seconds.

#### Participants

Twenty-three observers participated in Experiment 4. One participant’s data were excluded from the analysis based on the exclusion criteria.

#### Results

With respect to identifying the presence of a meaningful interaction, participants achieved a modest performance level with mean accuracy of 0.69 (*SD* = 0.15), significantly better than chance (*t*(21) = 5.77, *p* < .001), though considerably less than perfect. The modest level of performance in distinguishing partnered salsa dance couples from un-partnered dancer pairs is consistent with findings in a recent study [38]. Hence, the identification of interpersonal interactions in the present experiment was not trivial, but rather required effort and focused attention.

**Fig 6:**
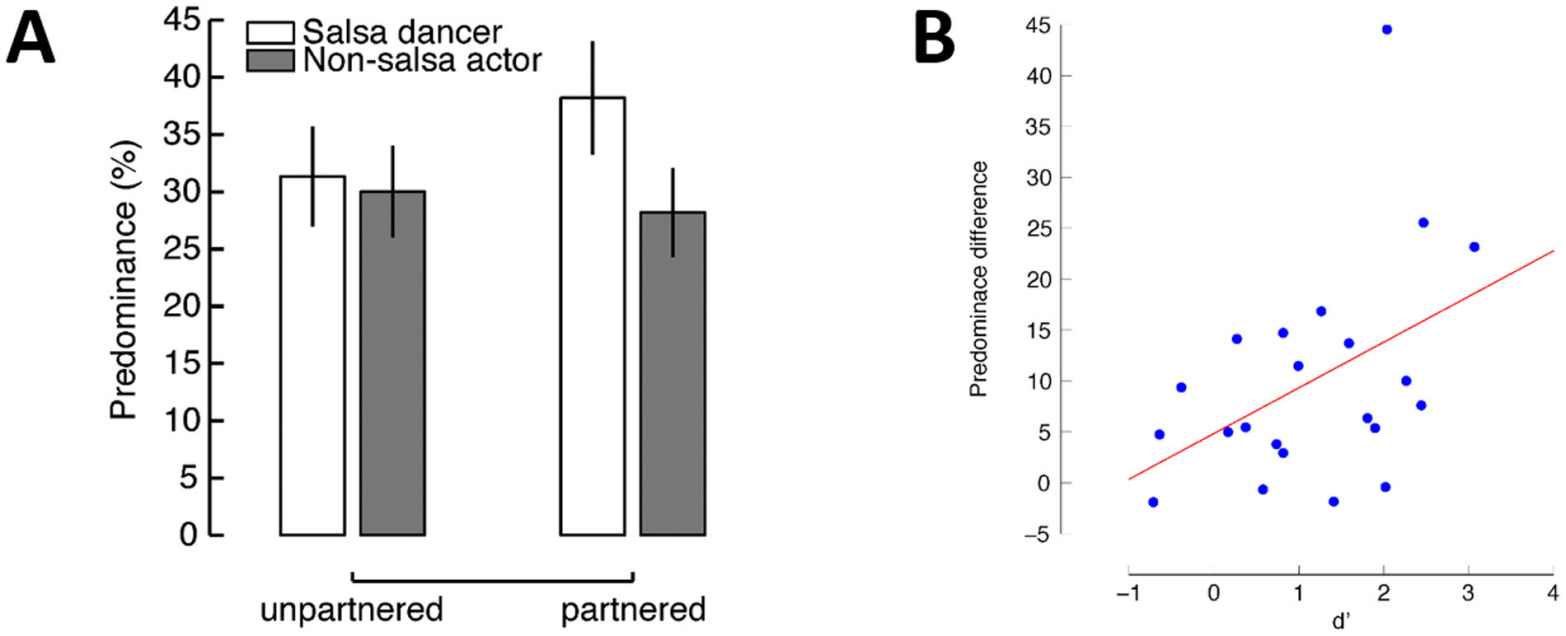
Results of Experiment 4: interactivity promotes visual dominance. (A) The predominance difference between the two rival actions was significantly greater in the presence of a partnered dancer than of an un-partnered dancer. Error bars indicate the 95% confidence intervals. (B) Scatter plot showing the relationship between predominance difference between the two rival actions in the partnered condition and sensitivity (d’) in identifying interactive activities.

In this experiment, we found that attentional focus on identifying meaningful social interactions significantly enhanced the visibility of the rival salsa dancer. As depicted in Fig 6A, the predominance of the rival salsa dancer increased when the partnered dancer was present compared to its rival non-salsa actor (salsa dancer, *M* = 38.21%; non-salsa actor, *M* = 28.20%, *t*(21) = 4.38, *p* < .001, Cohen’s *d* = .94). In contrast, the same comparison in the un-partnered condition did not yield any difference (salsa dancer, *M* = 31.34%; non-salsa actors, *M* = 30.00%, *t*(22) = 1.39, *p* = 0.180). The difference in predominance between the two rival actors thus depended on whether the third actor is was a partnered dancer or an un-partnered dancer performing similar dance movements (*F*(1,21) = 11.11, *p* = .003, $\eta_{p}^{2}$ = .35). We found that the predominance of the rival salsa dancer significantly increased in the presence of a meaningful interaction (partnered, 38.17%; un-partnered, 31.31%; *t*(21) = 3.03, *p* = .006, Cohen’s *d* = .61). Consistently, longer dominance durations were also obtained for the rival salsa dancer with interaction than without (partnered, 4.32 s; un-partnered, 3.69 s; *t*(21) = 2.22, *p* = .038, Cohen’s *d* = .19). As for the non-salsa action, predominance for was reduced slightly with interaction compared to without (partnered, 28.18%; un-partnered, 29.98%; *t*(21) = 2.55, *p* = .019, Cohen’s *d* = .54). However, this was not true for dominance durations (partnered, 3.82 s; un-partnered, 3.63 s; *t*(21) = 0.61, *p* = .546).

We further analyzed the relationship between predominance in the rivalry task and sensitivity in the identification task. The predominance difference between the two rival actions (i.e. the rival male salsa dancer and the non-salsa actor) in the partnered condition significantly correlated with sensitivity (d’) in identifying interactive activities (*r* = .44, *p* = .040, see Fig 6B; removing the potential outlier did not affect these data *r* = .45, *p* = .043). This finding suggests a positive association between ability in identifying social interactions and selectivity in promoting socially-relevant information to visual awareness. We also examined the potential relation between rivalry performance and response bias. We found that the subjective bias favoring the response of “interactive” did not correlate with enhanced visibility of interactive actors in the rivalry task (*r* = -0.12, *p* = .610). In addition, un-partnered trials which were misidentified as containing interactive activity showed no difference between the two rival actions in predominance (*t*(21) = 1.51, *p* = 0.147) or mean dominance duration (*t*(21) = 0.64, *p* = 0.528), excluding the possibility that participants merely reported the salsa dancer as dominant as long as they reported interactivity. Both results suggest that a response bias is unlikely to account for our main findings.

It might be argued that people have a bias to report or perceptually force the rival salsa dancers to be more visible because of its similarity with the binocularly-viewed female dancer (as both belong to the same action category, and were also task-relevant during the entire experiment). If this were the case, a similar effect would be expected in the un-partnered condition, since the binocularly-viewed dancer was a male salsa dancer selected from a different dancing couple thus showing even higher similarity with the rival male salsa dancer. However, we found equal predominance for the two rival actions in the un-partnered condition, ruling out this type of response bias as the cause of our finding.

## General Discussion

In the present study we found that actions eliciting natural body movements and meaningful interpersonal activities are granted preferential access to visual awareness. We employed binocular rivalry between natural unfamiliar activities, that contained a social interactions or not. Our approach contrasts with previous studies on rivalry of socially-relevant stimuli in the following important way: We investigated how social content in terms of *inter-personal interactions* influences the formation of visual awareness for human actions, whereas previous studies used non-interactive social stimuli (faces or isolated actors).

Previous studies that have used faces as social stimuli often employed continuous flash suppression (CFS) paradigm [39], a potent masking paradigm similar to binocular rivalry. It has been found that upright faces break into visual awareness more quickly than inverted faces [40], and some emotional faces break through especially quickly [41]. However, it has been argued that such effects may be due to low-level feature differences between rival stimuli [42], rather than the facial aspects per se (but see [43]).

Crucially, in the present study we controlled for low-level stimulus differences, and ensured that the rival actions were equally dominant without social interactions. These rival actions with equal stimulus strength made it possible to scrutinize how social interactions influence which action stimuli receive priority to conscious perception, and which action stimuli are suppressed as invisible. For rival actions that were equally visible in isolation (i.e., without the social context), we found that the inclusion of social interactions for one of rival actions significantly enhanced visibility of that action.

Our results on social interaction are reminiscent of previous studies showing contextual influences in binocular rivalry for simple grating stimuli (e.g., [15]). In that study, surrounding grating stimuli influenced the dominance balance of a pair of rivalry gratings. Analogously, the social contextual cues in interpersonal interactions impacted the rivalry dynamics in our stimuli. Furthermore, we found that interactive activities only increased the dominance durations of the action eliciting the inter-personal interactions, and did not affect the dominance duration of the non-interacting actor. This finding is consistent with what is known from binocular rivalry with grating stimuli [14], where it has been shown that the dominance duration of gratings increased if the rival grating was consistent with the context, while the dominance duration of the inconsistent stimulus remained unchanged. Experiments on similar simple stimuli also showed that attention only impinges on the dominance of the attended stimulus, and does not affect (i.e. suppress) the dominance of the non-attended stimulus [44]. We speculate that the same mechanisms are at work in our stimuli, with both contextual social information and attention being capable of increasing the dominance of the context-congruent stimulus, but leaving the context-incongruent stimulus largely unaffected.

Understanding actions, and social interactions in particular, requires an elaborate analysis of the visual input, including the context in which the actions take place. There is increasing evidence for a dual-processing strategy for understanding social actions and interactions. According to this theoretical framework, the first system analyzes social stimuli in an automatic manner, without the need for attentional focus, mainly through the mirror neuron system [34, 37, 45]. Even in the task of person recognition, a recent study [46] showed that humans automatically make use of body information to identify other individuals. This “automatic” system may rely on an embodied or “mirrored” simulation of the other’s actions to interpret observed activities or understand the underlying goals [35, 36]. These types of actions may include simple locomotion, such as walking, or simple goal-directed actions, such as reaching. The second system supports controlled social causal attribution, and is voluntarily engaged when a deeper understanding of actions is required [47]. The function of this so-called mentalizing system [48] can be impaired by high cognitive load or enhanced by attentional focus [37]. Interestingly, fMRI studies [7, 49] have shown that brain networks involved in the mirror and mentalizing systems are concurrently active when observing social interactions.

The findings in the present study are consistent with and further support the dual-process account. In the first three experiments, we showed that meaningful social (inter)actions gain preferential access to consciousness without requiring focused attention to social interactions (i.e. when the interaction is task-irrelevant). This effect is plausibly due to an automatic action-analyzing mechanism (presumably the mirror-neuron system). In Experiment 4 we showed that performing an attentional task specifically increased the access of social actions to consciousness. In a pilot experiment without the second task, we did not find the impact of interactivity (with partnered dancer vs. un-partnered dancer) on visual dominance of rival salsa dancer, suggesting that the attentional task is required. More critically, individual differences in the ability to identify interactive activity in complex scenes were correlated with increases in access of social actions to consciousness. It has previously been shown that attention impinges on the mentalizing system and not (or less so) on the mirror-neuron system [37], suggesting that the increase in the visual consciousness of interactive actors in Experiment 4 was likely due to an increased engagement of the mentalizing system. These findings extend previous research on individual differences in action perception [21, 50, 51] by suggesting an explicit link to the mentalizing system. By comparing the results in the last three experiments, we conclude that the involvement of automatic vs. attentional effects will depend on the specific comparison conditions in the experiment. If the comparison involves comparing the presence versus the absence of a cooperative dancer, the impact of social interactions is likely initiated automatically, because this is a relatively easy comparison (c.f. Experiment 2 and 3). However, when the comparison involves comparing subtle differences in interactivity (e.g., partnered dance couple vs. two actors performing similar dance movements; Experiment 4), attention engagement to social interaction is critical to actively modulate the visual dominance of the relevant action.

Binocular rivalry suppression is often thought to occur through interoccular inhibitory interactions between monocular channels [22, 52] that determine which stimulus reaches awareness (the dominant stimulus) and which stimulus does not (the suppressed stimulus). Our results show that social cues have a major impact on which visual information is gated to visual awareness, by exerting a large influence on interocular suppression, probably via cortical feedback [53, 54]. Our findings therefore imply that in controlling what stimuli can reach awareness, the human brain gives precedence to subtle socially relevant stimuli over non-social stimuli, very likely through contributions of both the mirror-neuron system and the mentalizing system. This selectivity to social cues enables exquisite sensitivity to socially relevant information, even before the visual stimuli reach full consciousness, especially when the visual inputs are noisy and ambiguous and competing for awareness or attention resources. Social cues are thus in the vanguard of information reaching conscious awareness, and that is made available for purposeful executive functions such as planning and contemplation [55], thereby enabling us to function effectively and efficiently in the complex social world in which we live.

## Supporting Information

S1 Fig The average inter-frame speed (pixel/frame) of joint movements for rival actions used in the Experiments 2 and 3. Error bars represent standard deviations.
(TIF)
