# Supplementary figures and images for: Social Interactions Receive Priority to Conscious Perception
Source: PLoS One. 2016 Aug 10;11(8):e0160468. doi: 10.1371/journal.pone.0160468 (PMC4980019; doi:10.1371/journal.pone.0160468)

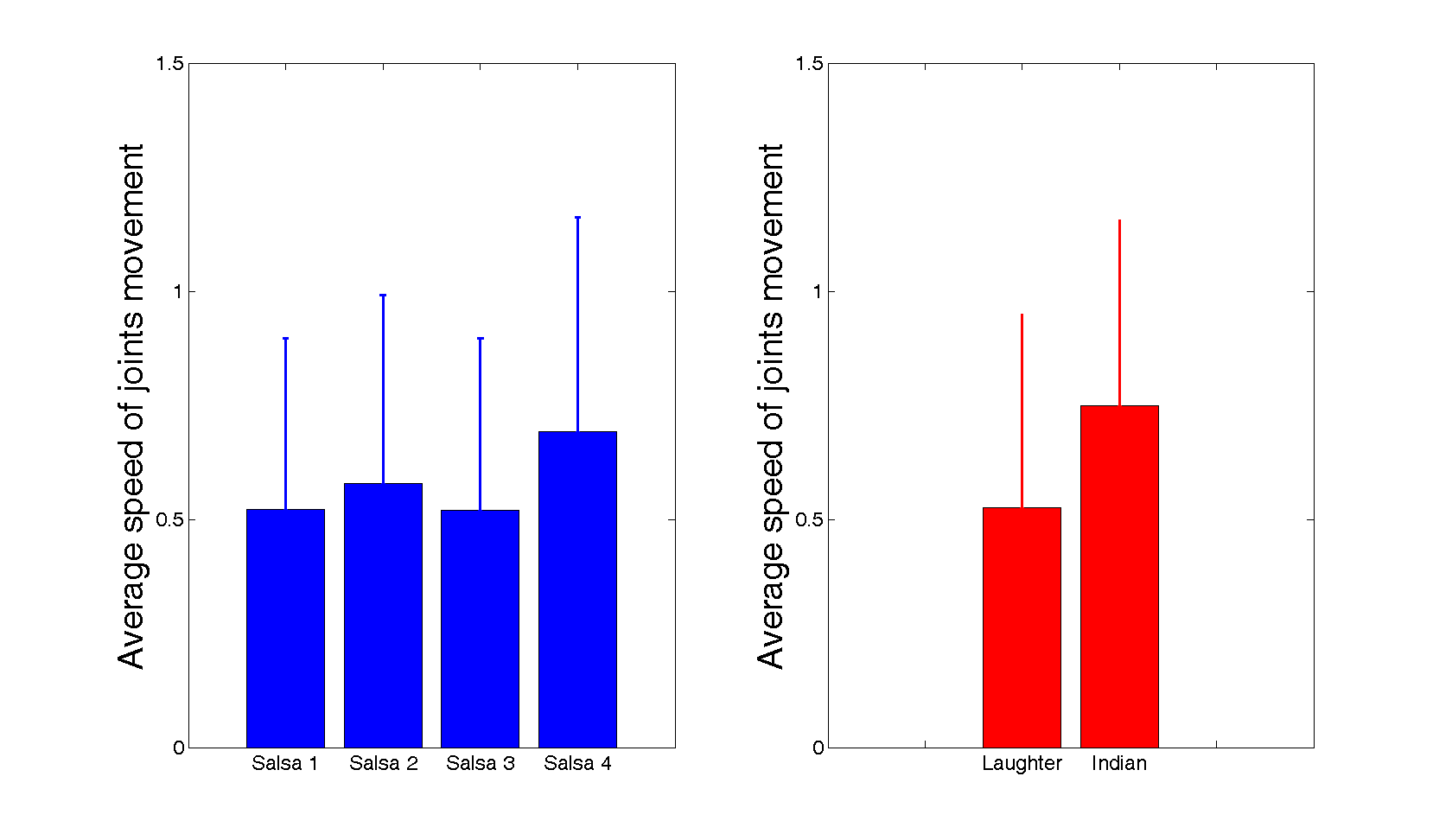

Supplement: S1 Fig — Error bars represent standard deviations. (TIF) [file pone.0160468.s001.tif]
